# Biomarkers of Tumor Metastasis and Invasiveness

**DOI:** 10.3390/cancers15205000

**Published:** 2023-10-16

**Authors:** Daniel L. Pouliquen, Cristina Núñez González

**Affiliations:** 1 Inserm, CRCI^2^NA, CNRS, Université d’Angers, Nantes Université, F-49000 Angers, France; 2 Research Unit, Lucus Augusti University Hospital (HULA), Servizo Galego de Saude (SERGAS), 27002 Lugo, Spain

The identification of proteins as new cancer diagnostic and prognostic biomarkers continues to attract considerable attention in the oncology literature, especially in the context of invasion and metastasis activation process [1]. In this field, the most recent developments include, but are not restricted to, proteins linked to the epithelial-to-mesenchymal transition (EMT), extracellular vesicles [2], co-receptors for growth factors [3], cell surface receptor adaptor proteins [4], transcription factors [5], or scaffolding proteins [6]. In this Special Issue, 169 authors representing 98 affiliations from 18 countries over 4 continents have made 17 contributions, and it is a great honor and pleasure for the Editors to introduce this collective work which summarizes important insights in this field of research.

In link with previous findings on EMT [2], Carrasco-Garcia et al. reported that a member of the SOX transcription factors (encoded by *SRY*-related HMG-box genes) that bind to the minor groove in DNA, SOX9, and linked to stem cell activity exhibited an increased expression in both pancreatic ductal adenocarcinoma cell lines and human biopsies. This observation was associated with metastasis, poor prognosis, and resistance to therapy [7]. Subsequently, previously reported biomarkers of effective adjuvant chemotherapy of non-small-cell lung cancer were reviewed by Tozuka and colleagues. They showed that cytoskeletal protein actinin-4, (ACTN4), previously reported to induce EMT through upregulation of a transcriptional repressor of E-cadherin, Snail, represented a possible biomarker for identifying patients at high risk of postoperative recurrence [8]. An in silico gene expression analysis, conducted by Rao et al., also established that, among 760 genes examined from the PANCAN Cancer Genome Atlas, a novel association was observed between the expression of vasoactive intestinal peptide (VIP) in gastrointestinal cancers and ZEB1-mediated EMT [9].

EMT has also been associated with mitochondrial dysfunction, which regulates the tumor microenvironment, leading to more aggressive tumors. Wu and colleagues reviewed the recent progress in the regulation of cancer metastasis by mitochondrial DNA, showing its impact on different aspects, including resistance to anoikis, promotion of angiogenesis, cancer cell survival in the circulatory system, and colonization [10]. Moreover, mitochondrial dysfunction leads to metabolic reprogramming and proteome rewiring. In their cross-species investigation of mitochondrial metabolic differences between invasive and non-invasive mesothelioma of four experimental rat tumor models and ten patient-derived cell lines, Pouliquen et al. showed that the most impressive expression increase concerned one enzyme of the fatty acid oxidation, the long-chain acyl coenzyme A dehydrogenase (ACADL) [11].

Other components of the tumor microenvironments also influence EMT. In extrahepatic cholangiosarcoma, another type of aggressive tumor, Oba et al. found an association between one chemokine receptor, CCR7, which is primarily expressed in various immune cells, and EMT in human cell lines. Interestingly, their clinicopathological examination also revealed that high-grade CCR7 expression was one of the most adverse postoperative prognostic factors in patients undergoing surgical resections of this tumor and was associated with more tumor buds and mesenchymal status [12]. The involvement of immune tumor microenvironment in clinical management was also documented in osteosarcoma by Huang and colleagues. They highlighted the role and mechanism of three proteins associated with the immune infiltration and progression of this cancer type, BCL2 interacting protein 3, prostaglandin I2 synthase, and the adhesion plaque protein Zyxin [13], all of which having been previously individually classified as unfavorable prognosis biomarkers (https://www.proteinatlas.org/, accessed on 29 August 2023). Among immune cells, the role played by the infiltration of gastric cancer by lymphocyte subsets, combined with the Prognostic Nutritional Index, was also emphasized by Sun and colleagues. Their study revealed the peculiar role of CD19+ B cells to identify patients with a high risk of metastasis and recurrence after surgery [14].

For some aggressive types of cancers such as glioblastoma multiforme (GBM), since 2021, the grading includes molecular features of the tumor. To improve its characterization, the search for new diagnosis biomarkers has led to investigation of the protein cargo of extravesicles in the blood and cerebrospinal fluid, highlighting some potential candidates [15]. However, further work is required to translate the basic research into clinical practice. To date, a few molecular biomarkers have shown potential to predict the survival outcomes and treatment response in patients, with some limitations [16]. As other biomarkers need to be investigated, in this Special Issue, Marshland et al. revealed the clinical relevance of sortilin, a membrane receptor involved in the sorting and transporting of intracellular proteins, which represents a potential biomarker and therapeutic target for GBM [17].

Like sortilin, other proteins circulating in the blood of patients with invasive cancers were recently investigated. In a large study conducted on 3272 patients with non-small-cell lung cancer (NSCLC), Jiang and colleagues explored the roles of seven proteins to predict tumor metastasis and stage. They found that patients exhibiting combined increased levels of three serum tumor markers, carcinoembryonic antigen, cytokeratin-19 fragment, and carbohydrate antigen 199 tended to have higher tumor stages, while the two latter were indicative of lymphatic and distant metastasis [18]. In their combined analysis of blood plasma and bone marrow mononuclear cells proteomes from patients with multiple myeloma, Dunphy et al. also revealed that an aggressive, extramedullary form of this disease was associated with a significant differential abundance of three promising biomarkers, vascular cell adhesion molecule 1, pigment epithelium-derived factor, and hepatocyte growth factor activator [19].

Another field of investigation is represented by enzymes secreted into the extracellular spaces. One example was provided by the work of Chen and Chai on prostasin and matriptase, two membrane serine proteases showing opposite effects in solid epithelial tumors, that reciprocally activate each other. In B lymphoma cells, they explored the utility of prostasin exosomes in matriptase activation to initiate a prostasin–matriptase activation cascade and analyzed its impact on the invasive properties of different cell lines [20]. As remodeling of the extracellular matrix plays an important role in tumor progression, among zinc-dependent proteases, a growing interest concerns the A disintegrin and metalloproteinases family (ADAM), their involvement in gastrointestinal tumor progression being reviewed by Lukaszewicz-Zajac et al. They highlighted the promising significance of seven members of this family as potential prognostic biomarkers and therapeutics targets for these cancers [21].

Distant metastasis represents a major problem for invasive cancers, which frequently concerns lymph nodes. In this field, attempts to identify biomarkers of lymph node invasion are continuously produced, which have led, for example, to recently highlighting the role played by CD47 in the progression of colorectal cancer [22]. In the case of high-grade endometrial cancer, Lombaers et al. reported that the increased risk of lymph node metastasis observed in patients with stage III–IV disease was associated with elevated levels of cancer antigen 125 (CA125) and reduced overall survival, confirming its predictive value [23]. In patients with lung adenocarcinoma, Yu and colleagues revealed that the expression of one member of the mitogen-activated protein kinase family, MAPK15, was positively correlated with lymph node metastasis. Moreover, mechanistically, MAPK15 was shown to interact with p50 to promote the expression of the prostaglandin E2 receptor EP3 subtype at the transcriptional level, thereby enhancing cancer cell migration and metastasis [24]. In patients with squamous cell carcinoma (SCC) of the skin, Klein et al. reported that the high expression of the insulin-like growth factor 2 mRNA-binding protein 3 (IMP3) observed in 122 cases with high-risk localizations (lip, ear) was correlated with aggressiveness features, including lymph node metastases [25]. Finally, for another primary tumor site of this cancer, in the tongue tissue, Casili and colleagues used an in vivo orthotopic model of oral SCC to demonstrate the therapeutic potential of an inhibitor of one member of the nucleotide-binding domain leucine-rich repeat-containing receptors (NLRs), playing essential roles in immunity and inflammation. This protein, NOD-like receptor protein 3 (NLRP3), for which the activation of induces the assembly of multiprotein complexes known as inflammasomes, represents a potential prognostic biomarker for different cancer types [26,27]. They showed that treatment with this molecule modulated EMT in the tongue tissue as well as in metastatic organs such as lymph nodes [28].

In conclusion, we hope that this Special Issue will attract readers interested in this crucial topic in basic cancer research, which could help generate important future biomedical applications for the benefits of patients.
